# Assessment of the performance of assertive community treatment: the case of Bizkaia (Spain)

**DOI:** 10.1192/j.eurpsy.2022.870

**Published:** 2022-09-01

**Authors:** D. Díaz-Milanés, N. Almeda, C. García-Alonso, L. Salvador-Carulla

**Affiliations:** 1 Universidad Loyola Andalucía, Psychology, Seville, Spain; 2 Universidad Loyola Andalucía, Quantitative Methods, Seville, Spain; 3 University of Canberra, Faculty Of Health, Canberra, Australia

**Keywords:** assertive community treatment, relative technical efficiency, Monte-Carlo simulation, Mental Health Policy

## Abstract

**Introduction:**

A mental health (MH) assertive community treatment (ACT) is always designed expecting for a decrease in the pressure (visits and readmissions) in inpatient services and to increase care quality. An appropriate management of ACT provision can be crucial to develop a balanced community-based MH ecosystems.

**Objectives:**

To assess the impact of the ACT on the performance of the MH ecosystem of Bizkaia (Basque Country, Spain).

**Methods:**

The ecosystem is structured by 19 MH areas, supported by 5 ACT teams. Here ACT provides high intensity mobile outpatient care to people suffering from severe mental disorders. The impact of these teams on the ecosystem performance was assessed by Monte-Carlo simulation, the Data Envelopment Analysis (DEA) and fuzzy inference. The input variables were the availability, number of psychiatrics, nurses and total of professionals of ACT services in each area. The outputs were: frequentation, incidence and prevalence of ACT services in each MH area. Performance indicators were: relative technical efficiency (RTE), statistical stability and entropy.

**Results:**

The global ecosystem performance was high (RTE on average=0.799 -input DEA orientation- and 0.825 -output orientation- up to 1, the maximum), the stability was medium-low (respectively 38,67% and 13.64% up to 100%, the maximum) and the entropy was medium-high (respectively 70,41% and 65.9% up to 100%, the maximum).

**Conclusions:**

Results highlighted a positive impact of ACT in Bizkaia. Nevertheless, stability and entropy levels showed the existence of a high structural variability in ACT services due to the necessity of adjusting them to the user’s specific needs.

**Disclosure:**

No significant relationships.

